# Neighborhood walkability and 12-year changes in cardio-metabolic risk: the mediating role of physical activity

**DOI:** 10.1186/s12966-019-0849-7

**Published:** 2019-10-15

**Authors:** Manoj Chandrabose, Ester Cerin, Suzanne Mavoa, David Dunstan, Alison Carver, Gavin Turrell, Neville Owen, Billie Giles-Corti, Takemi Sugiyama

**Affiliations:** 1 Mary MacKillop Institute for Health Research, Australian Catholic University, Melbourne, Australia; 2 Centre for Urban Transitions, Swinburne University of Technology, Melbourne, Australia; 3 Baker Heart and Diabetes Institute, Melbourne, Australia; 4 School of Public Health, The University of Hong Kong, Hong Kong, China; 5 Melbourne School of Population and Global Health, University of Melbourne, Melbourne, Australia; 6 Centre for Urban Research, RMIT University, Melbourne, Australia; 7 School of Public Health and Social Work, Queensland University of Technology, Brisbane, Australia; 8 School of Public Health, The University of Queensland, Brisbane, Australia; 9 Central Clinical School, Faculty of Medicine, Nursing and Health Sciences, Monash University, Melbourne, Australia; 10 Institute for Resilient Regions, University of Southern Queensland, Toowoomba, Australia

**Keywords:** Built environment, Cardiovascular disease, Type 2 diabetes, Hypertension, Pathways, Population health

## Abstract

**Background:**

Living in walkable neighborhoods may provide long-term cardio-metabolic health benefits to residents. Little empirical research has examined the behavioral mechanisms in this relationship. In this longitudinal study, we examined the potential mediating role of physical activity (baseline and 12-year change) in the relationships of neighborhood walkability with 12-year changes in cardio-metabolic risk markers.

**Methods:**

The Australian Diabetes, Obesity and Lifestyle study collected data from adults, initially aged 25+ years, in 1999–2000, 2004–05, and 2011–12. We used 12-year follow-up data from 2023 participants who did not change their address during the study period. Outcomes were 12-year changes in waist circumference, weight, systolic and diastolic blood pressure, fasting and 2-h postload plasma glucose, high-density lipoprotein cholesterol, and triglycerides. A walkability index was calculated, using dwelling density, intersection density, and destination density, within 1 km street-network buffers around participants’ homes. Spatial data for calculating these measures were sourced around the second follow-up period. Physical activity was assessed by self-reported time spent in moderate-to-vigorous physical activity (including walking). Multilevel models, adjusting for potential confounders, were used to examine the total and indirect relationships. The joint-significance test was used to assess mediation.

**Results:**

There was evidence for relationships of higher walkability with smaller increases in weight (*P* = 0.020), systolic blood pressure (*P* < 0.001), and high-density lipoprotein cholesterol (*P* = 0.002); and, for relationships of higher walkability with higher baseline physical activity (*P* = 0.020), which, in turn, related to smaller increases in waist circumference (*P* = 0.006), weight (*P* = 0.020), and a greater increase in high-density lipoprotein cholesterol (*P* = 0.005). There was no evidence for a relationship of a higher walkability with a change in physical activity during the study period (*P* = 0.590).

**Conclusions:**

Our mediation analysis has shown that the protective effects of walkable neighborhoods against obesity risk may be in part attributable to higher baseline physical activity levels. However, there was no evidence of mediation by increases in physical activity during the study period. Further research is needed to understand other behavioral pathways between walkability and cardio-metabolic health, and to investigate any effects of changes in walkability.

## Background

Due to the increasing global burden of cardio-metabolic diseases, such as type 2 diabetes (T2D) and cardiovascular disease, urgent preventive action has been advocated [1]. In addition to individual-level approaches to reducing risk factors, greater attention is now being given to community-level approaches that address the contextual factors where people live [2]. A growing body of research has examined the role of the built environment in cardio-metabolic disease prevention [3–6]. A recent review of longitudinal studies found that residents living in higher walkability neighborhoods (characterized by high residential density, mixed land use, and high street connectivity) are less likely to develop obesity, T2D, and hypertension over time, compared with those who live in lower walkability neighborhoods [3]. Environmental initiatives to reduce cardio-metabolic disease risk are promising as they are likely to have sustained effects at the community level [7].

It is important to identify behavioral pathways that may underlie the relationships between the built environment and cardio-metabolic disease [3–7]. This could inform the development of effective environmental and policy initiatives for targeting chronic disease prevention [7]. Physical activity is a strong candidate for mediating these relationships. Neighborhood environmental attributes including walkability are associated with residents’ physical activity levels [8–11], and regular participation in physical activity reduces cardio-metabolic disease risk [12–14]. However, existing studies examining the mediating role of physical activity in the relationships between walkability and cardio-metabolic health have in most part focused on cross-sectional associations with obesity-related outcomes [15–17]. The findings of those studies suggest indirect associations between walkability and obesity-related outcomes through physical activity. In order to further advance our understanding, it is important to examine how physical activity, which may change over time, accounts for the long-term health benefits of neighborhood walkability [3]. Further, it is known that active lifestyles can be effective in improving other cardio-metabolic health profiles (blood pressure, blood glucose, and blood lipids), independent of their effects on obesity-related measures [18]. Thus, research needs to further examine the potential mediating effects of physical activity in the relationship of walkability with multiple markers of cardio-metabolic disease.

Three longitudinal studies have examined the mediating role of physical activity in relationships between walkability and cardio-metabolic health outcomes [19–21]. Two tested mediation by using the Barron and Kenny’s approach [22], examining the attenuation in the relationship between walkability and cardio-metabolic health by comparing regression coefficients before and after adjusting for physical activity [20, 21]. This approach, however, is not in line with recent advances in methods of mediation analysis [23, 24]. Indeed, tests of mediation based on the Barron and Kenny’s approach have been found to provide incorrect findings [25, 26]. Further, this approach relies on the total effect (direct and through all possible mediating pathways) of the exposure on the outcome being statistically significant in order to assess mediation (indirect) effects. However, it is now recognized that an indirect effect of the exposure on the outcome through mediators can exist even in the absence of a significant total effect (i.e., multiple opposite directional mediators exist and cancel each other out) [23, 24]. One recommended way to test mediating effects is to separately assess the effects of exposures on mediators and the exposure-adjusted effects of mediators on outcomes [23, 25]. An Australian study used this method to assess the mediating role of physical activity measured at a single time point in the relationship of walkability with 10-year changes in glycosylated hemoglobin (HbA1c, a marker of cardio-metabolic disease) and found a partial mediation effect [19]. However, the mediating role of physical activity change in the relationship of walkability with residents’ cardio-metabolic health over time has not been examined.

**Fig. 1:**
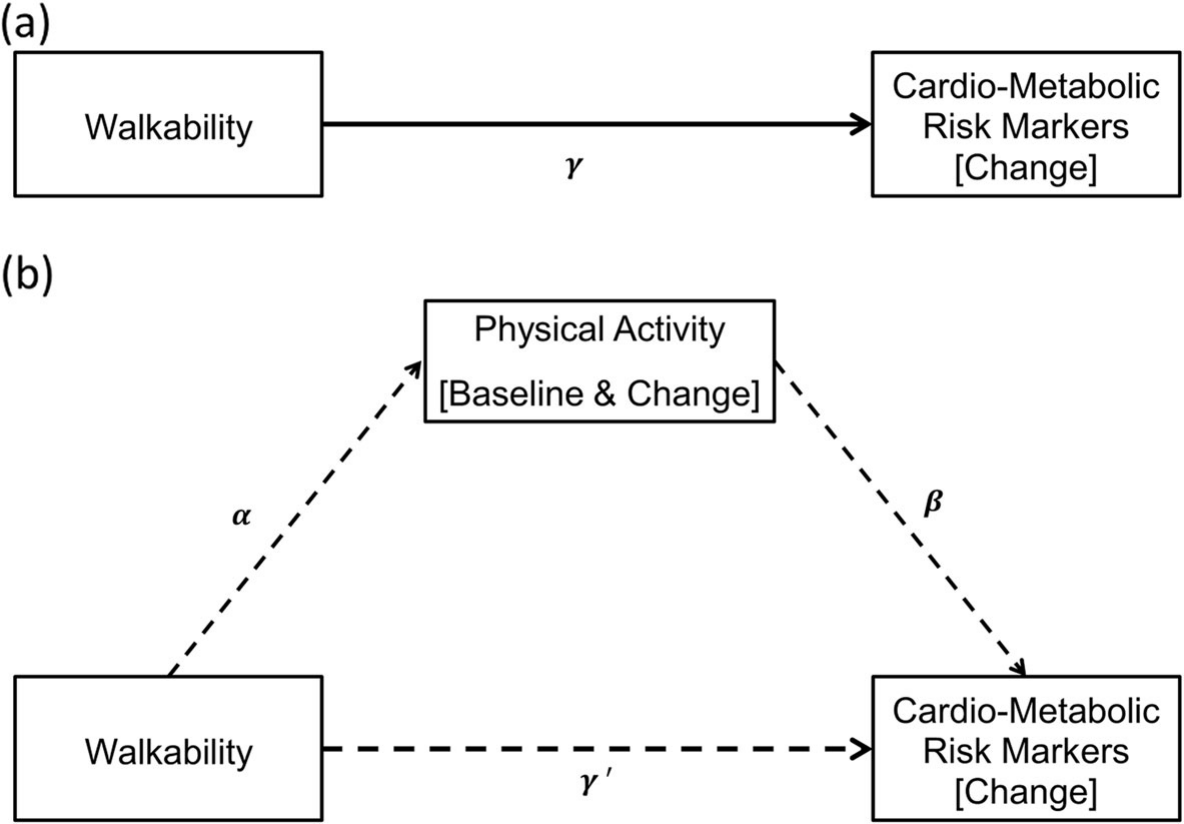
Relationships of walkability with changes in cardio-metabolic risk markers (**a**), mediated through the baseline and the change in physical activity (**b**)

The aims of our study were twofold: first, to examine the total effects of neighborhood walkability on 12-year changes of cardio-metabolic risk markers (estimating *γ* in Fig. 1a); second, to examine the indirect effects of neighborhood walkability on changes in the outcomes, mediated through physical activity at baseline and changes in physical activity (estimating *α* and *β* in Fig. 1b). We hypothesized that high walkability would be protective against increasing cardio-metabolic risk over time, and that those protective effects would be partly attributable to high baseline levels and subsequent increases in physical activity.

## Methods

### Data source

Data were from the Australian Diabetes, Obesity and Lifestyle Study (AusDiab), which is an Australian national longitudinal cohort study [27]. The primary aim of AusDiab is to examine the prevalence and determinants of obesity, diabetes, and cardiovascular disease. AusDiab collected survey and biomedical data in three waves: baseline in 1999–2000 (AusDiab1), first follow-up in 2004–05 (AusDiab2), and second follow-up in 2011–12 (AusDiab3). Details about the AusDiab1 study design and recruitment procedures have been published elsewhere [27]. Briefly, a two-stage stratified cluster sampling design was used to select 42 study areas in the metropolitan and regional cities of six states and the Northern Territory. From each study area, a random sample of adults (aged 25 years and over, with no physical or intellectual disabilities, and residing at their addresses for 6 months or longer prior to the survey) was selected. A study area consisted of contiguous Census Collector District (CCD) geographical area units. A CCD was the smallest area unit for the collection of Census data at the time of AusDiab1, averaging approximately 225 dwellings [28]. In total, 11,247 participants provided both survey and biomedical data in AusDiab1 (response rate = 55.3%). From the baseline cohort, 6400 (retention rate = 59.3%) and 4614 (retention rate = 44.6%) participants provided both survey and biomedical data in AusDiab2 and AusDiab3, respectively. The International Diabetes Institute and the Alfred Hospital Ethics Committee approved the study (approval no. 39/11). All participants provided written informed consent to participate in the study.

### Study participants

Our sample consisted of participants for whom data were available over 12 years. There were 3968 who provided data at all three observation points, and 646 who provided data for AusDiab1 and AusDiab3 only. Of these, we excluded those whose addresses were not accurately geocoded (*N* = 81) and who moved residence during the study period (*N* = 2140). The reason for excluding movers was that it is unknown for how long they were exposed to different neighborhoods between observation points since their relocation date was not recorded. Further, we excluded 15 participants who reported being pregnant during data collection; 151 who reported that they had coronary heart disease or stroke prior to or during the study period; 209 who reported difficulties in walking more than 500 m at any of three observation points; and 11 who were older than 78 years at baseline [29] (numbers are not mutually exclusive). The reason for excluding these subgroups was to reduce possible reverse causality bias, as their health status may have had stronger influences on their physical activity behaviors during the study period [30]. The final analytical sample size was 2023.

### Outcome variables

The outcomes examined were annual changes in cardio-metabolic risk markers over 12 years: waist circumference (WC), body weight (weight), systolic blood pressure (SBP), diastolic blood pressure (DBP), fasting plasma glucose (FPG), 2-h postload plasma glucose (2-h PG), high-density lipoprotein cholesterol (HDL-C), and triglycerides (TG). These markers were measured at local data-collection centers at each time point. The details of the measurement methods and instruments used were described previously [27].

### Exposure variables

The primary exposure variable of our study was a neighborhood walkability index. The walkability index typically consists of measures of residential density, street connectivity, and land use mix [31]. Given the difficulty of obtaining nationally consistent fine-scale land use data for calculating land use mix (entropy) measures in Australia, Mavoa et al. [32] developed an alternative measure using access to daily living destinations. Following their method, we created a walkability index using residential density, street connectivity, and daily living destinations. They were calculated for each participant within a 1 km street-network buffer (sausage-type, with 150 m radius from street centerline) around their residential location [33]. We chose a 1 km buffer to represent residential neighborhoods because this distance was shown to be a typical distance within which most neighborhood walking trips by adults take place [34]. In the current study, it was not possible to obtain retrospective spatial data, for calculating walkability, that corresponds to the baseline of the study (1999–2000). We, thus, sourced spatial data around the second follow-up period. The details of each of the walkability components are given below. ArcGIS v.10.6 (ESRI, Redlands) was used for geographic information system (GIS) data processing and spatial analysis.

#### Residential density

Residential density was defined as the number of dwellings within the buffer divided by its area. The dwelling count data in the mesh blocks (smallest census geographical units) were obtained from the Australian Bureau of Statistics 2011 Census [35] to calculate an individual buffer based dwelling density measure [36].

#### Street connectivity

We used intersection density as the measure of street connectivity. Intersection density was defined as the number of 4-or-more way intersections within the buffer divided by its area, which has previously been shown to be associated with walking in the context of Australia [37]. Road network data from PSMA Australia’s 2012 Transport & Topography dataset were used to calculate this measure.

#### Daily living destinations

Access to daily living destinations was measured as the density (total count divided by buffer area) of different types of neighborhood destinations to which residents may travel daily/regularly: supermarkets, convenience stores, and public transport stops. This destination-based measure was developed in Australia to assess land use diversity at the national scale, and found to be correlated with an entropy measure of land use mix and associated with walking for transport [32]. Axiom Business Points data and Supermarkets data from Pitney Bowes Ltd. (sourced in 2013) were used to obtain locations of convenience stores and supermarkets. PSMA Australia’s 2012 Transport data were used to obtain locations of railway stations for commuters. General Transit Feed Specification online repository data (http://transitfeeds.com, sourced in 2015) were used to obtain locations of tram stops. Bus stops were not used in this study because their inclusion inflates this measure (about 90% of participants had at least one bus stop and over 25% of participants had 25+ bus stops within 1 km buffer).

#### Walkability index

A walkability index was calculated by standardizing (z-score) the summed standardized measures of residential density, intersections density, and daily living destinations density.

### Mediating variables

Participant’s self-reported time spent in physical activity was used to obtain the two potential mediator variables. At each wave of the AusDiab study, participants were asked to report the time they spent in a range of physical activities during the previous week using the Active Australia Survey (AAS) [38, 39]. The items used were shown in Additional file 1: Material S1. Total time (mins per week) was calculated as the sum of the time spent in walking (for recreation and transport), moderate-intensity physical activity, plus double the time spent in vigorous-intensity physical activity [39]. The AAS instrument has been shown to have acceptable levels of reliability and validity for the measure of weekly total physical activity duration among adults [40, 41]. To avoid measurement error due to over-reporting, we truncated the weekly total physical activity duration at 1680 min (28 h) per week following the AAS procedure [39]. Total time at AusDiab1 was used as the baseline measure. To estimate the annual change in physical activity, we calculated the 12-year change in physical activity geometrically, as shown in Additional file 1: Material S2. This method allowed us to incorporate all three time points in assessing the change in physical activity. This is superior to a simpler method of subtracting the baseline value from the 12-year follow-up value, which disregards the 5-year follow-up value and assumes a constant change throughout the study period.

### Potential confounders

We included the following variables (assessed at baseline) as potential confounders: gender, age, education, marital status, employment status, household income, household children status (having a child or children in the household), and height (for weight only). Since a change in participants’ socio-demographic status over time may influence their long-term physical activity and cardio-metabolic health profiles, we also included changes (from baseline to wave 3) in marital status, employment status, household income, and household children status as potential confounders of the relevant longitudinal models. For instance, change in employment status was classified as: kept working, stopped working, started working, or not working. Further, hypertension medication use (for SBP and DBP only), medication/insulin treatment for diabetes or family history of diabetes (for FPG and 2-h PG only), and cholesterol medication use (for HDL-C and TG only) were included as potential confounders in the relevant models. These variables are defined as binary variables (yes: participant was on medication in at least one of the three observation points; no: participant was not on medication at any observation point). For area-level socioeconomic status, we used the Index of Relative Socioeconomic Disadvantage (IRSD), which is a census-based composite variable consisting of measures such as income, education, employment, household structure, and car ownership [42]. The 2011 IRSD scores corresponding to the Australian Standard Geographical Classification’s Statistical Local Area (SLA) units were used. For the study areas that sat across multiple SLAs, the mean IRSD value was employed (12 study areas sat across two SLAs and one study area sat across three SLAs).

### Statistical analyses

#### Calculating changes in cardio-metabolic risk markers

We used multilevel unconditional linear growth models to estimate each participant’s annual change in cardio-metabolic risk markers by utilizing measures at three observation points [43]. Briefly, for each risk marker, repeated measures (of individuals who were nested within study sites) were modeled with the time at which the corresponding measure was obtained as a predictor. We used continuous time metrics: t = 0 for AusDiab1 (baseline); t = 5 for AusDiab2 (5-year follow-up); and t = 12 for AusDiab3 (12-year follow-up). These multilevel (three-level) growth models included random intercepts at the participant and the study area level and random slopes for time metrics at the participant level. Inclusion of random intercepts for participants allowed those observations from the same participants (repeated measures) to be correlated, and inclusion of random intercepts for study areas allowed those participants living in the same areas (participants recruited from pre-selected CCDs) to be correlated. Participant-specific random intercept and random slope of time metric (corresponding to participant’s linear trajectory line) estimated the starting point and the annual change of the risk marker, respectively [see Additional file 1: Material S2]. An unstructured covariance matrix was specified between participant-specific random intercepts and random slopes to allow them to correlate. The point estimate of the regression coefficient of time represents the annual change for the average participant.

#### Examining the total effects

To examine the total effect of the walkability index on changes in cardio-metabolic risk markers (corresponding to *γ* in Fig. 1a), the above described multilevel linear growth models were extended by adding the walkability index and other potential confounders as participant-level and area-level predictors (see Additional file 1: Material S3 for further details) [44].

#### Testing mediation

To test mediation, we estimated regression coefficients *α* and *β* in Fig. 1b. For *α*, we used a two-level generalized linear mixed model with a Gamma distribution and log link function to examine the relationship of the walkability index with baseline physical activity (right-skewed); and a two-level linear mixed model with a Normal distribution and identity link function to examine the relationship of the walkability index with changes in physical activity (Normally distributed). In both models, random intercepts were included at the study area level to account for area-level clustering. The model for baseline physical activity was adjusted for the baseline socio-demographic variables only; while the model for change in physical activity was adjusted for both baseline and change in socio-demographic variables, and baseline physical activity. For *β*, the above described multilevel linear growth models to estimate the total effects were extended by further adding the baseline and changes in physical activity along with the walkability index and potential confounders. To assess the statistical significance of the mediating effect, we used the joint-significance test [26], in which simultaneous significance of the regression coefficients *α* and *β* provides evidence for mediating effects.

#### Missing data and loss to follow-up

In multi-level linear growth models, for each risk marker outcome variable, all participants with at least a baseline measurement for the corresponding marker were included in the analyses. Multilevel modeling of repeated measures over time assumes missing at random (MAR) mechanism for missing data, implying that missingness can be ignored if all variables related to attrition are included in the model [44].

Statistical analyses were performed in STATA (v.15.0) and R (v.3.5.0).

## Results

Table 1 shows the characteristics of the study sample. The mean follow-up duration was 11.9 years (range: 11.0 to 12.4 years). The comparison of baseline characteristics of those included in the current study (stayers), excluded (movers), and who withdrew from the AusDiab study is shown in Additional file 1: Table S1. Compared with those who provided 12-years follow-up data, movers were more likely to be younger and not living with a partner, while drop-outs were more likely to be older, less educated, had lower income levels, not working, not living with a partner or children, had poorer health profiles and having lower physical activity levels at baseline.

**Table 1:** Baseline characteristics of study participants, AusDiab study, 1999–2000, (*N* = 2023)

Baseline characteristics	Means (SD) or Percentages
Age, years	49.8 (10.2)
Gender, % Women	54.5
Education	
% High school or less	33.1
% Technical or vocation	43.1
% Bachelor’s degree or more	23.8
Employment status	
% Working	74.3
% Not working	25.1
% Others	0.6
Weekly household income	
% Less than $600	27.4
% $600–1500	48.0
% > $1500	24.7
Marital status, % couple	86.1
Children in household, % yes	48.3
Cardio-metabolic risk markers	
WC (cm)	88.7 (13.1)
Weight (kg)	75.5 (15.4)
SBP (mmHg)	127.0 (16.7)
DBP (mmHg)	70.4 (11.3)
FPG (mg/dL)	98.7 (17.6)
2-h PG (mg/dL)	107.3 (35.6)
HDL-C (mg/dL)	55.9 (14.6)
TG (mg/dL)	127.8 (87.4)
Total physical activity (hours/week)	5.0 (6.1)
Walking (hours/week)	2.1 (2.7)
Moderate-intensity physical activity (hours/week)	1.0 (2.7)
Vigorous-intensity physical activity (hours/week)	0.9 (2.0)
Medication use (reported at least at one wave)	
For hypertension, % yes	32.1
For type 2 diabetes (including insulin), % yes	4.8
For high cholesterol, % yes	23.5
Family history of diabetes (pooled across waves), % yes	29.0
Index of Relative Socioeconomic Disadvantage (2011 Census)	1021.4 (58.6)

Abbreviations: *WC* Waist Circumference, *SBP* Systolic Blood Pressure, *DBP* Diastolic Blood Pressure, *FPG* Fasting Plasma Glucose, *2-h PG* 2-h Postload Plasma Glucose, *HDL-C* High-Density Lipoprotein Cholesterol, *TG* Triglycerides

Table 2 shows descriptive statistics for the walkability index and its components, and Pearson’s correlation coefficients between each pair of them. Correlation coefficients between walkability components ranged from 0.4 to 0.6.

**Table 2:** Descriptive statistics for walkability and its components within participants’ 1 km street-network residential buffers, AusDiab study, 1999–2012, (*N* = 2023)

Walkability components	Mean (SD)	Min	Q1	Median	Q3	Max	Correlation Matrix			
							Res. density	Int.density	Des.density	Walkability
Res. density ^a^	7.1 (3.6)	0.1	4.5	6.6	9.4	26.2	1.0	0.6*	0.4*	0.8*
Int. density ^b^	4.0 (4.5)	0.0	0.8	2.3	5.2	20.7		1.0	0.4*	0.8*
Des. density ^c^	1.5 (1.5)	0.0	0.0	1.2	2.3	8.1			1.0	0.7*
Walkability	0.0 (1.0)	−1.6	−0.7	− 0.2	0.6	5.1				1.0

Abbreviations: *Res* Residential, *Int* Intersections, *Des* Destinations;

*P < 0.001

^a^Number of dwellings/hectare within 1 km of each residence

^b^Number of 4-way intersections/km^2^ within 1 km of each residence

^c^Number of daily living destinations/km^2^ within 1 km of each residence

Table 3 shows the mean change from AusDiab1 to AusDiab3 and the mean annual change (estimated from the unconditional growth models) of each cardio-metabolic risk marker. Overall, on average, participants increased their WC, weight, blood pressure, and glucose levels, but improved their lipid profiles over the 12-year period. The mean (SD) weekly total physical activity duration at baseline was 5.0 (6.1) hours/week and its mean change over the 12-year study period was 1.2 (9.3) hours/week (i.e., increase).

**Table 3:** Mean changes cardiometabolic risk markers, AusDiab study, 1999–2012, (*N* = 2023)

Cardiometabolic Risk markers	No of participants included in models	Mean (95% CI) change from AusDiab1 to 3	Mean^a^ (95% CI) annual change
WC (cm)	2023	5.35 (5.02, 5.67)	0.45 (0.42, 0.47)
Weight (kg)	2019	2.25 (1.95, 2.54)	0.18 (0.16, 0.21)
SBP (mmHg)	2019	3.00 (2.25, 3.74)	0.30 (0.24, 0.36)
DBP (mmHg)	2019	2.20 (1.66, 2.74)	0.20 (0.16, 0.25)
FPG (mg/dL)	2023	−0.08 (− 0.93, 0.77)	0.01 (− 0.06, 0.08)
2-h PG (mg/dL)	1997	1.97 (0.39, 3.56)	0.15 (0.02, 0.29)
HDL-C (mg/dL)	2023	3.58 (3.12, 4.05)	0.31 (0.27, 0.35)
TG (mg/dL)	2023	−10.24 (− 13.54, −6.94)	−0.87 (− 1.15, − 0.6)

Abbreviations: *WC* Waist Circumference, *SBP* Systolic Blood Pressure, *DBP* Diastolic Blood Pressure, *FPG* Fasting Plasma Glucose, *2-h PG* 2-h Postload Plasma Glucose, *HDL-C* High-Density Lipoprotein Cholesterol, *TG* Triglycerides

^a^Estimated from the unconditional growth model

Table 4 shows the results of regression models examining the total effects of walkability index on annual changes in cardio-metabolic risk markers (*γ* regression coefficients). After adjusting for potential confounders, there was evidence for relationships of higher walkability index with smaller annual increases in weight (*P* = 0.028), SBP (*P* < 0.001), and HDL-C (*P* = 0.002); and there was also some weaker evidence for relationships of higher walkability index with smaller annual increases in WC (*P* = 0.092) and FPG (*P* = 0.053).

**Table 4:** Total effects of walkability index on annual changes in cardio-metabolic risk markers, AusDiab study, 1999–2012, (*N* = 2023)

Cardio-metabolic risk marker	*γ*- regression coefficients (95%CI)	*P*-value
WC (cm)	−0.02 (− 0.05, 0.00)	0.092
Weight (kg)	−0.03 (− 0.05, 0.00)	**0.028**
SBP (mmHg)	−0.15 (− 0.21, − 0.08)	**< 0.001**
DBP (mmHg)	0.01 (− 0.03, 0.05)	0.552
FPG (mg/dL)	−0.06 (− 0.13, 0.00)	0.053
2-h PG (mg/dL)	0.01 (− 0.11, 0.14)	0.826
HDL-C (mg/dL)	−0.06 (− 0.10, − 0.02)	**0.002**
TG (mg/dL)	0.04 (− 0.18, 0.26)	0.702

Abbreviations: *WC* Waist Circumference, *SBP* Systolic Blood Pressure, *DBP* Diastolic Blood Pressure, *FPG* Fasting Plasma Glucose, *2-h PG* 2-h Postload Plasma Glucose, *HDL-C* High-Density Lipoprotein Cholesterol, *TG* Triglycerides

Models adjusted for baseline age, gender, education, baseline work status, baseline household income, baseline marital status, baseline household children status, changes in socio-demographic factors (work status, household income, marital status, and household children status), height (only for weight), hypertension medication use (for SBP and DBP only), treatment for diabetes and family history of diabetes (for FPG and 2-h PG only), cholesterol medication use (for HDL-C and TG only), and Index of Relative Socio-economic Disadvantage. Regression coefficients correspond to 1 SD increment in walkability index. *P*-value < 0.05 in boldface

With regard to the associations of walkability index with the baseline and the annual change in physical activity (*α* coefficients), after adjusting for potential confounders, there was evidence for the relationship of higher walkability index with higher baseline physical activity (exp(*α*) [95% CI] = 1.09 [1.01, 1.16], *P* = 0.020); but not with the annual change in physical activity (*α* [95% CI] = 0.01 [− 0.03, 0.05] hours/week, *P* = 0.590).

Table 5 shows the results of regression models examining the effects of the baseline and the annual change in physical activity on annual changes in cardio-metabolic risk markers (*β* regression coefficients). After adjusting for walkability index and other potential confounders, there was evidence for relationships of higher baseline physical activity with smaller increases in WC (*P* = 0.006), weight (*P* = 0.020), and a greater increase in HDL-C (*P* = 0.005). In the corresponding regression models, there was evidence for relationships of an increase in physical activity related with smaller increases in WC (*P* < 0.001), weight (*P* = 0.005), DBP (*P* = 0.050), FPG (*P* = 0.019), TG (*P* = 0.004), and a greater increase in HDL-C (*P* < 0.001).

**Table 5:** Relationships of the baseline and the annual change in physical activity with annual changes in cardio-metabolic risk markers, adjusted for walkability index, AusDiab study, 1999–2012 (*N* = 2023)

Cardio-metabolic risk markers	*β−* regression coefficients			
	Baseline physical activity (hours/week)		Change in physical activity (hours/week)	
	*β* (95%CI)	*P*-value	*β* (95%CI)	*P*-value
WC (cm)	−0.008 (− 0.014, − 0.002)	**0.006**	−0.096 (− 0.139, − 0.053)	**< 0.001**
Weight (kg)	−0.006 (− 0.011, − 0.001)	**0.020**	−0.056 (− 0.094, − 0.017)	**0.005**
SBP (mmHg)	−0.001 (− 0.013, 0.012)	0.926	0.023 (− 0.070, 0.116)	0.624
DBP (mmHg)	−0.004 (− 0.011, 0.004)	0.372	−0.058 (− 0.116, 0.000)	0.050
FPG (mg/dL)	−0.005 (− 0.016, 0.006)	0.382	−0.099 (− 0.181, − 0.016)	**0.019**
2-h PG (mg/dL)	−0.011 (− 0.038, 0.015)	0.397	−0.155 (− 0.354, 0.044)	0.126
HDL-C (mg/dL)	0.012 (0.004, 0.020)	**0.005**	0.158 (0.095, 0.221)	**< 0.001**
TG (mg/dL)	−0.028 (− 0.074, 0.018)	0.236	− 0.516 (− 0.863, − 0.169)	**0.004**

Abbreviations: *WC* Waist Circumference, *SBP* Systolic Blood Pressure, *DBP* Diastolic Blood Pressure, *FPG* Fasting Plasma Glucose, *2-h PG* 2-h Postload Plasma Glucose, *HDL-C* High-Density Lipoprotein Cholesterol, *TG* Triglycerides. Models adjusted for walkability index, baseline age, gender, education, baseline work status, baseline household income, baseline marital status, baseline household children status, changes in lifestyle factors (work status, household income, marital status, and household children status), height (only for weight), hypertension medication use (for SBP and DBP only), treatment for diabetes and family history of diabetes (for FPG and 2-h PG only), cholesterol medication use (for HDL-C and TG only), and Index of Relative Socio-economic Disadvantage. *P*-value < 0.05 in boldface

## Discussion

This study examined the total effects of neighborhood walkability on cardio-metabolic risk changes over 12 years, and whether physical activity mediated these relationships. Below, we first discuss our findings on the total effects, mediation by physical activity (baseline and change), followed by limitations and strengths.

### Total effects

For the total effect of walkability on cardio-metabolic risk markers, we found evidence that higher walkability index was related to smaller increases in weight and related to smaller increases in WC (weaker evidence). These findings suggest that living in high walkable areas may be protective against the development of obesity. We observed that one standard deviation (SD) higher walkability index was related to smaller annual weight gain by 0.03 kg (Table 4). Considering that the mean annual weight gain for this sample was 0.18 kg (Table 3), the total effect of one SD higher walkability on residents’ weight gain was around 17%, which can be interpreted as being a substantial effect at the population level [45]. A recent systematic review of longitudinal studies found strong evidence for a protective effect of higher walkability against the development of obesity [3]. Our study thus contributes to this growing evidence base, which suggests that initiatives to improve neighborhood walkability could make an important contribution to reducing the burden of obesity [46].

For blood pressure markers, we found that a higher walkability index was related to smaller increases in SBP, but not DBP. A recent study conducted in the UK also reported similar findings [47]. Further, the finding on the effect of higher walkability on SBP change was also consistent with two studies conducted in the USA [48, 49]. For blood glucose markers, we found that higher walkability index was related to smaller increases in FPG, but not with 2-h PG. Other studies have also produced mixed findings for relationships of walkability with changes in T2D risk markers [19, 48, 50]. The systematic review of longitudinal studies found strong evidence for potential protective effects of higher walkability against the development of hypertension and T2D [3]. Our current findings partly support the beneficial relationship of walkability with blood pressure and blood glucose found in existing studies. For blood lipid markers, we found that higher walkability index was related to a smaller increase in HDL-C, but not with TG. Notably, the relationship between walkability and HDL-C was in the unexpected direction (living in a high walkable neighborhood leading to poorer blood lipid profiles). This finding is, to some extent, consistent with a previous longitudinal study conducted in the USA that found a greater increase in TG for those who moved to higher walkability neighborhoods from lower walkability neighborhoods [50]. A recent systematic review of mostly cross-sectional studies also found less favorable blood lipid levels among urban residents as compared with rural residents [51]. These inconsistent or unexpected findings may be due to other potentially relevant exposures not measured in this study, such as easier access to unhealthy food outlets [52], which may have some detrimental effects on blood pressure, glucose, and lipids. Future research might consider examining the spatial co-location of walkability and other environmental exposures to investigate their independent and joint relationships with cardio-metabolic disease risk.

### Mediation by baseline physical activity

Based on the joint-significance test, we found evidence suggesting that baseline physical activity mediates the relationship between walkability and changes in obesity-related measures (i.e., higher walkability index was related with higher baseline physical activity, which predicted smaller annual increases in WC and weight). This finding is consistent with previous cross-sectional studies on mediation by physical activity in the relationship between walkability and obesity [15–17], using mediation analysis methods similar to those used in this study. However, our study extends the previous findings by showing the mediating role of physical activity in the long-term protective effect of higher walkability against obesity. The mediation analysis also found that higher baseline physical activity, which was related to higher walkability, had a beneficial impact on cholesterol. This is contradictory to the observed total effect, where higher walkability led to adverse cholesterol changes over time. It is possible that higher walkability itself has positive effects on blood lipids through facilitating physical activity. But, as discussed above, walkable neighborhoods may also provide easy access to unhealthy food outlets [52]. The detrimental effects of greater energy intake may have outweighed the benefits provided by greater physical activity. This warrants further investigation. Research incorporating multiple relevant health behaviors is needed to understand the seemingly contradictory findings.

Notably, no evidence was found for relationships of physical activity measured at baseline with changes in blood pressure, blood glucose, and triglycerides. A possible explanation may be that in the context that physical activity changes over a longer follow-up period, the baseline physical activity may fail to predict the long-term beneficial health gains [53–55].

### Mediation by changes in physical activity

We also examined whether changes in physical activity levels over time may be a factor mediating the relationships between neighborhood walkability and changes in cardio-metabolic risk. Although physical activity changes were related to changes in most of the risk markers examined in the study, walkability (measure at a single time point) was not related to physical activity changes. Thus, according to the joint-significance test, physical activity changes may not be considered as a mechanism through which neighborhood walkability influences cardio-metabolic risk over time. A recent review on the longitudinal relationships of built environments with physical activity reported that environmental attributes measured at one point of time may not contribute to changes in physical activity [10]. People’s behavior choice is known to be habitual, often triggered by environmental cues [56]. Given that this study focused on participants who stayed in the same residence, it is possible that increasing physical activity may require additional non-environmental stimuli, such as advice from health professionals, new incentives to use active modes of travel, and social pressure to exercise. Natural experimental studies examining changes in environments (due to relocation or environmental modification) are needed to explore the mediating role of physical activity changes in the environmental impacts on cardio-metabolic health. It is possible that the behavioral changes observed are attributable to environmental changes, which we could not measure in this study.

### Limitations and strengths

Limitations of this study include the use of self-reported physical activity measures: measurement error may have resulted in incorrect estimations. The association observed between walkability and baseline physical activity may be confounded by self-selection of neighborhoods [57]. Neighborhood walkability is more closely related to transport-related walking [58], which is typically lower in intensity than exercise. However, inclusion of leisure-time physical activity and exercise may have contributed to weakening the relationship between walkability and total physical activity. Future research needs to examine the role of physical activity in specific domains and intensity levels. The attrition rate was relatively high due to the longer follow-up period (55%). Under the assumption of MAR mechanism, up to 60% loss to follow-up was less likely to produce biased estimates of effects [59]. However, if attrition was “missing not at random” (i.e., loss to follow-up depends on the outcome variable), the estimated effects may have been biased and led to invalid conclusions [59]. We used a walkability index that was created based on geospatial data sourced around the time of AusDiab3. This was due to the unavailability of relevant data for the baseline period (1999–2000). It is possible that some study areas may have changed little, while others may have undergone further development during the study period [60]. Future longitudinal research may have to consider how baseline and change in walkability can influence residents’ cardio-metabolic risk.

Strengths of our study include sufficiently large sample size, longitudinal design with a 12-year follow-up period (three measurement points), the use of objective measures of cardio-metabolic risk markers, the use of GIS-based walkability measure, and a broad range of study areas from multiple urban settings across Australia. The study tested mediation following recent advancements in mediation analysis methods. We also used a sophisticated statistical method, multilevel growth model, in analyzing the complex data (repeated measures within individuals, who were recruited using stratified cluster sampling).

## Conclusions

Our findings suggest that neighborhood environments designed to encourage residents’ physical activity may help reduce the risk of obesity and related disease over time. Improving neighborhood walkability may be a potential strategy to enhance population health by encouraging more physical activity. Further studies are recommended to examine specific environmental attributes that may contribute to reducing cardio-metabolic risk (not only obesity but also hypertension, hyperglycemia, and hyperlipidemia) through physical activity. Such understanding would support policy-makers and practitioners in urban design and planning to develop healthier neighborhoods. Our study found an adverse effect of high walkability on blood lipids, suggesting the presence of other unhealthy exposures in high walkable areas. Research is needed to examine other behavioral pathways (e.g. diet) through which walkability may influence residents’ cardio-metabolic health.

## Supplementary information

**Additional file 1: Table S1.** Baseline characteristics of stayers, movers, and drop-outs, AusDiab study (1999-2012); Material S1. Active Australia Survey Items Used to Measure Physical Activity; Material S2. Calculating Changes Using Values Measured at Three Observation Points; Material S3. Details of the Three-Level Linear Growth Model Used in the Study  

## Data Availability

Data that support the findings of this study are available on request under a license agreement. Written applications can be made to the AusDiab Steering Committee (Dianna.Magliano@baker.edu.au).

## Abbreviations

2-h PG: 2-h Postload Plasma Glucose
AAS: Active Australia Survey
AusDiab: Australian Diabetes, Obesity and Lifestyle Study
CCD: Census Collector Districts
DBP: Diastolic Blood Pressure
FPG: Fasting Plasma Glucose
HDL-C: High-Density Lipoprotein Cholesterol
IRSD: Index of Relative Socioeconomic Disadvantage
MAR: Missing at Random
SBP: Systolic Blood Pressure
T2D: Type 2 Diabetes
TG: Triglycerides
WC: Waist Circumference
