# Supplementary material for: Neighborhood walkability and 12-year changes in cardio-metabolic risk: the mediating role of physical activity
Source: Int J Behav Nutr Phys Act. 2019 Oct 15;16:86. doi: 10.1186/s12966-019-0849-7 (PMC6792258; doi:10.1186/s12966-019-0849-7)
Supplement: Supplementary file 1 — Additional file 1: Table S1. Baseline characteristics of stayers, movers, and drop-outs, AusDiab study (1999-2012); Material S1. Active Australia Survey Items Used to Measure Physical Activity; Material S2. Calculating Changes Using Values Measured at Three Observation Points; Material S3. Details of the Three-Level Linear Growth Model Used in the Study [file 12966_2019_849_MOESM1_ESM.docx]

**Table S1**. Baseline characteristics of stayers, movers, and drop-outs, AusDiab study (1999-2012)

| Baseline characteristics | Mean (SD) or Percentage | | |
| --- | --- | --- | --- |
|  | Stayers  (N=2,023) | Movers  (N=2,164) | Drop-outs  (N= 6,633) |
| Age, years | 49.8 (10.2) | 46.8^ǂ^ (11.4) | 53.3^ǂ^ (16.1) |
| Gender, % Women | 54.5 | 56.7 | 55.0 |
| Education  % High school or less  % Technical or vocation  % Bachelor’s degree or higher | 33.1  43.1  23.8 | 32.3  44.3  23.4 | 46.3^ǂ^  41.0  12.8^ǂ^ |
| Employment status  % Working (incl. students)  % Not working  % Others | 74.3  25.1  0.6 | 76.2  23.5  0.3 | 50.7^ǂ^  48.9^ǂ^  0.5 |
| Weekly household income  % Less than $600  % $600-1500  % >$1500 | 27.4  48.0  24.7 | 29.1  48.7  22.2 | 12.4^ǂ^  36.5^ǂ^  51.2^ǂ^ |
| Marital status, % couple | 86.1 | 78.7^ǂ^ | 72.2^ǂ^ |
| Children in household, % yes | 48.3 | 46.8 | 34.7^ǂ^ |
| Cardio-metabolic risk markers  WC (cm)  Weight (kg)  SBP (mmHg)  DBP (mmHg)  FPG (mg/dL)  2-hr PG (mg/dL)  HDL-C (mg/dL)  TG (mg/dL) | 88.7 (13.1)  75.5 (15.4)  127.0 (16.7)  70.4 (11.3)  98.7 (17.6)  107.3 (35.6)  55.9 (14.6)  127.8 (87.4) | 89.2 (13.7)  76.4 (16.1)  124.7^ǂ^ (16.0)  69.0^ǂ^ (11.3)  98.2 (19.3)  107.1 (35.3)  55.9 (14.7)  126.7 (83.3) | 92.0 (14.2)^ǂ^  76.7 (16.6)^ǂ^  131.4 (19.9)^ǂ^  70.3 (12.0)  102.2 (23.8)^ǂ^  118.7 (47.2)^ǂ^  54.7 (15.0)^ǂ^  143.8 (100.8)^ǂ^ |
| Total physical activity (hours/week)  Walking (hours/week)  Moderate-intensity physical activity (hours/week)  Vigorous-intensity physical activity (hours /week) | 5.0 (6.1)  2.1 (2.7)  1.0 (2.7)  0.9 (2.0) | 5.0 (6.2)  2.0 (2.8)  0.9 (2.6)  1.0 (2.0) | 4.7 (6.2)^ǂ^  2.1 (2.9)  0.9 (2.5)  0.7 (2.0)^ǂ^ |
| Medication use (at baseline)  For hypertension, % yes  For type 2 diabetes (including insulin), % yes  For high cholesterol, % yes  Family history of diabetes (reported at baseline), % yes | 9.1  1.2  5.5  19.9 | 9.1  1.7  5.7  18.8 | 19.4^ǂ^  4.0^ǂ^  10.0^ǂ^  18.2 |

Note: WC, Waist Circumference; SBP, Systolic Blood Pressure; DBP, Diastolic Blood Pressure; FPG, Fasting Plasma Glucose; 2-hr PG, 2-hour Postload Plasma Glucose; HDL-C, High-Density Lipoprotein Cholesterol; TG, Triglycerides. In total, 11,247 participants were recruited at the baseline. Of those, 4,614 participants completed surveys and biomedical examination at the 12-years follow-up. After excluding participants whose addresses were not geocoded (N=81), there were 2,369 stayers and 2,164 movers. Among the stayers, those who reported pregnant during data collection, cardiovascular disease incidence, walking difficulties, and older than 78 years at baseline (N= 346, in total) were excluded in the current study. ^ǂ^ Significantly different (*P*<0.05) in comparison to stayers; *P*-values were obtained using t-test (for continuous variables) or Chi-squared test (for categorical variables).

**Material S1. Active Australia Survey Items Used to Measure Physical Activity**

**AusDiab: The Australian Diabetes, Obesity and Lifestyle Study**

**General Questionnaire**

**Physical activity**

In this section, I will ask you some questions about the time that you may have spent doing physical activities as part of your everyday lives.

***The following questions are about any physical activities that you may have done in the last week.***

**Walking**

In the last week, how many times have you walked continuously, for at least 10 minutes, for recreation, exercise or to get to or from places?

|  |  | times |
| --- | --- | --- |

What do you estimate was the total time that you spent walking in this way in the last week?

(In hours and/or minutes - fill in all circles on answer sheet)

|  |  | hours |  |  | minutes |
| --- | --- | --- | --- | --- | --- |

**Vigorous physical activity**

The next question does not include household chores, gardening or yard work.

In the last week, how many times did you do any vigorous physical activity which made you breathe harder or puff and pant? (e.g. tennis, jogging, cycling, keep fit exercises).

|  |  | times |
| --- | --- | --- |

What do you estimate was the total time that you spent doing this vigorous physical activity in the last week?

(In hours and/or minutes - fill in all circles on answer sheet)

|  |  | hours |  |  | minutes |
| --- | --- | --- | --- | --- | --- |

**Moderate physical activity**

The next question does not include household chores, gardening or yard work.

In the last week, how many times did you do any other more moderate physical activities that you have not already mentioned? (e.g. lawn bowls, golf, gentle swimming, etc.)

|  |  | times |
| --- | --- | --- |

What do you estimate was the total time that you spent doing these activities in the last week?

(In hours and/or minutes - fill in all circles on answer sheet)

|  |  | hours |  |  | minutes |
| --- | --- | --- | --- | --- | --- |

**Material S2. Calculating Changes Using Values Measured at Three Observation Points**

**Calculating the change in physical activity using** **values at all three observation points**


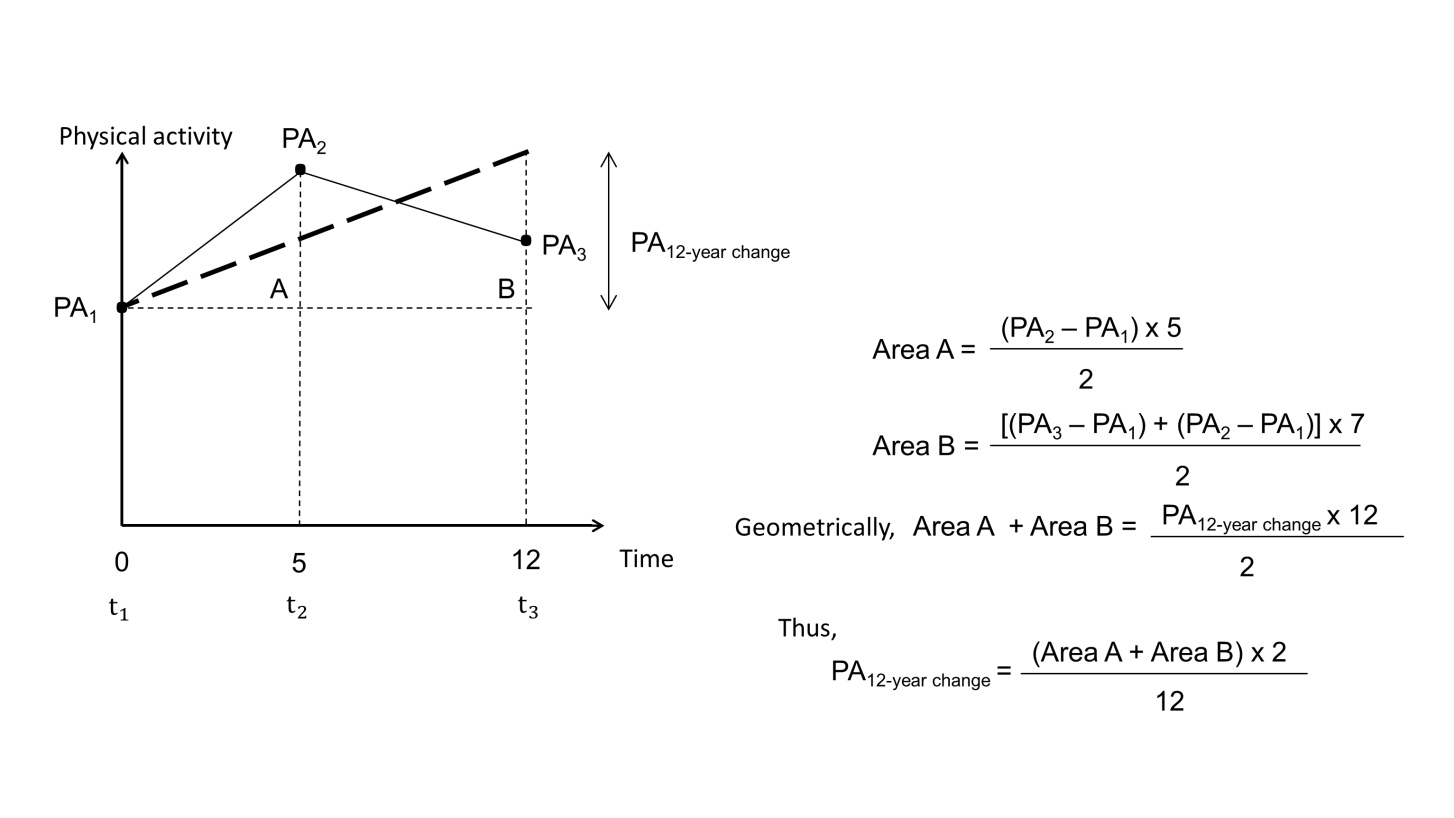


Figure S1: Calculating the change in physical activity (PA) using measures at all three observation points

**Example:** Suppose that a participant had values PA_1_ =150 mins/week, PA_2_ =300 mins/week, PA_3_=220 mins/week. The simple 12-year change in PA (calculated by subtracting the baseline value from the 12-year follow-up value) = 70 mins/week (thus, average annual increase = 5.8 mins/week). The12-year change in PA (calculated using values at all three observation points, as above) = 191 mins/week (thus, average annual increase = 16 mins/week).

**Calculating changes in cardio-metabolic risk markers using values at all three observation points**


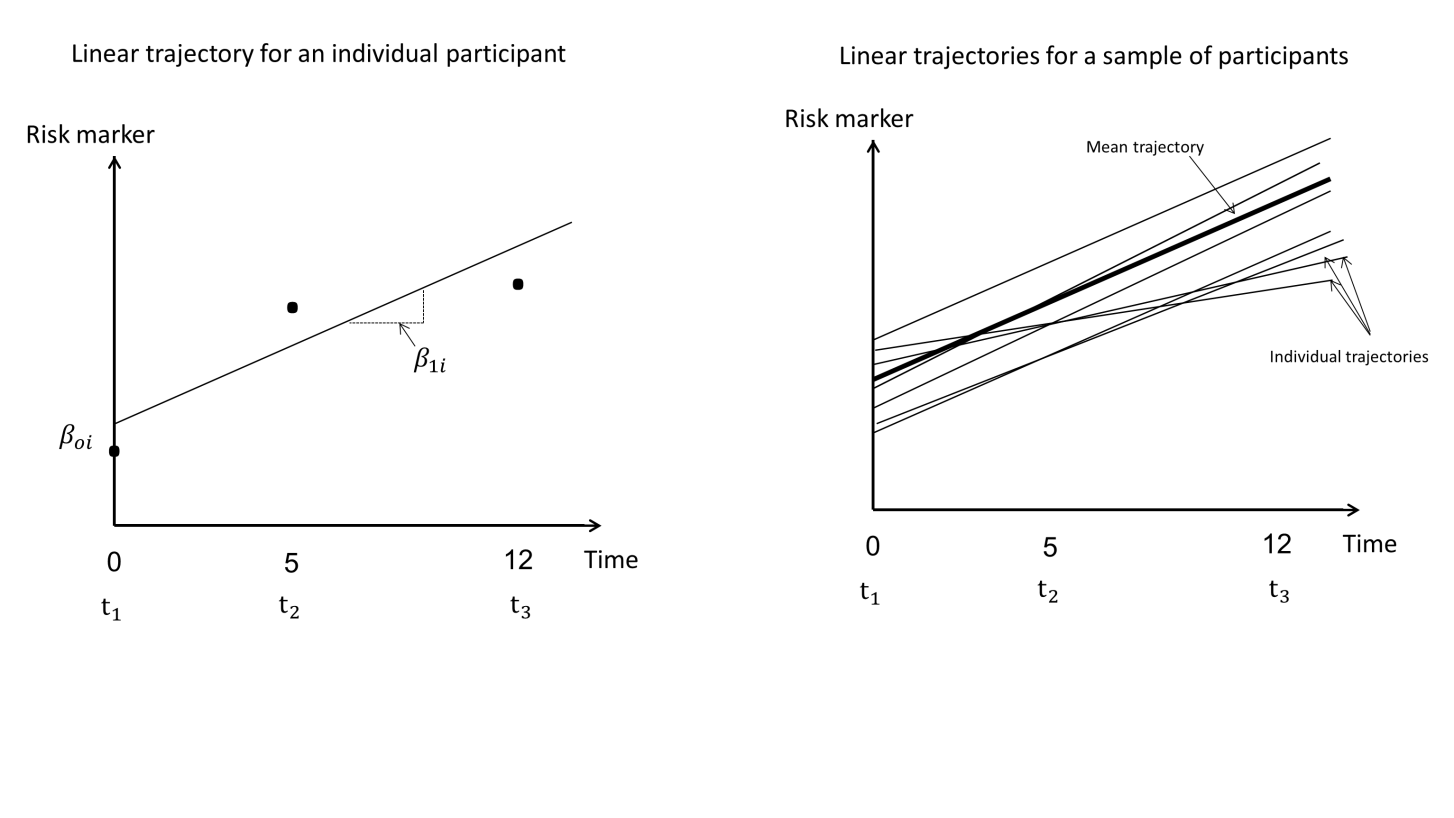


Figure S2: Estimating changes in cardio-metabolic risk markers using measures at all three observation points

**Material S3. Details of the Three-Level Linear Growth Model Used in the Study**

The data used in this study has a three-level structure: repeated observations are nested within study participants who are nested within study areas (Figure S3).


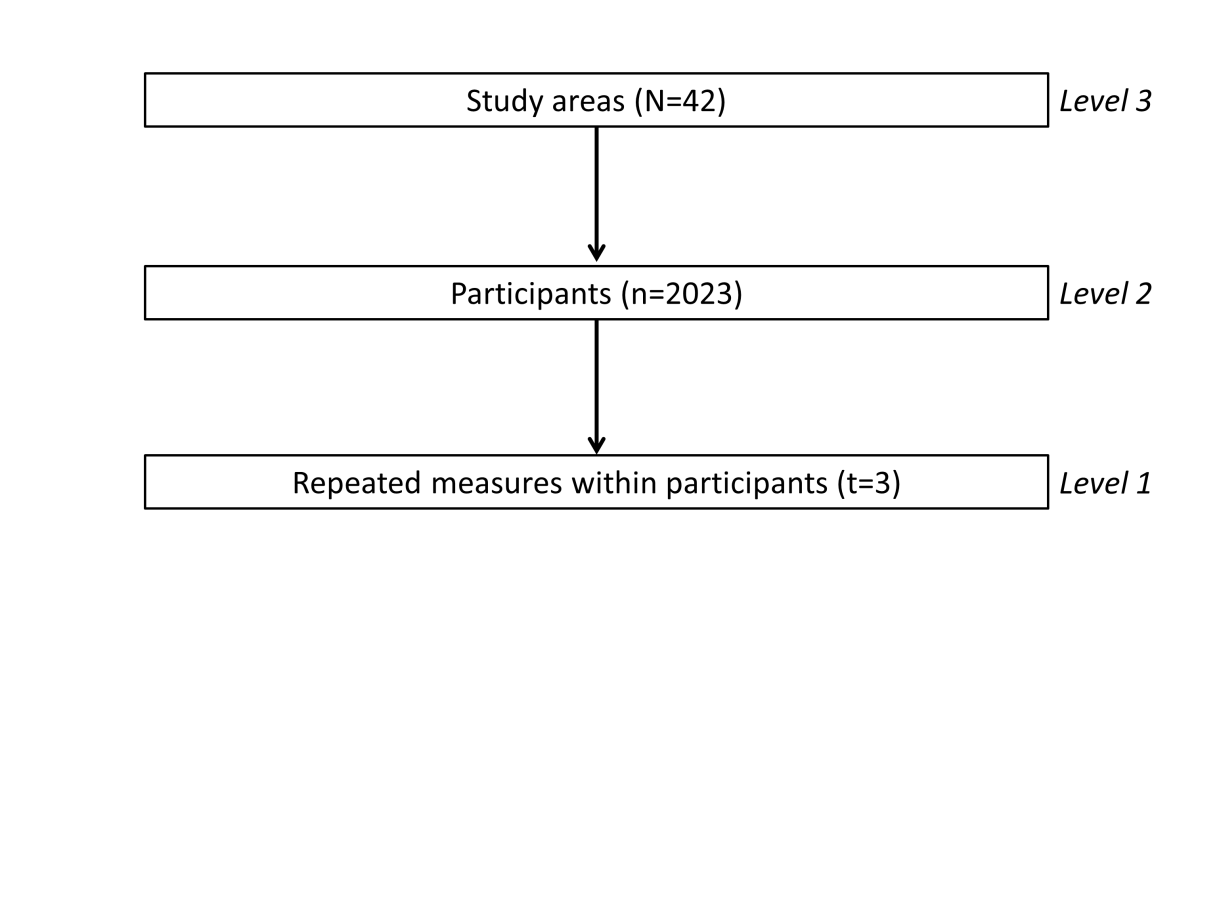


Figure S3: Three-level Structure of the Data

In this data structure, the cardio-metabolic risk marker was assigned at Level 1 (at most, three repeated measures for each participant). The participant-level exposure variable (i.e., walkability) and confounders were assigned at Level 2. The area-level confounder (i.e., area-level SES) was assigned at Level 3. The multilevel linear growth model was developed as*:*

- Level 1 (time-level) model $\Rightarrow$

$$y_{tij}=b_{0ij}+\boldsymbol{b}_{\boldsymbol{1}\boldsymbol{i}}t{+e}_{tij}$$

- Level 2 (participant-level) models $\Rightarrow$

$$b_{0ij}= \beta_{00j}+{\beta_{01} walkability}_{i}+{\beta_{02} (participant level confounders)}_{i} +u_{0ij}$$

$$\boldsymbol{b}_{\boldsymbol{1}\boldsymbol{i}}= \beta_{1}+{\boldsymbol{\beta}_{\boldsymbol{11}}\boldsymbol{walkability}}_{\boldsymbol{i}}+{\beta_{12} (participant level confounders)}_{i} +u_{1i}$$

- Level 3 (area-level) model $\Rightarrow$

$$\beta_{00j}= \gamma_{000}+{\gamma_{001}(area level confounders)}_{j} +v_{00j}$$

Here, $y_{tij}$ is the value of the risk marker measured at time point $t$ for study particpant $i$ who resides in study area $j$. The random intercept $b_{0ij}$is the mean of $y_{tij}$ across the time points for a particpant $i$ who resides in a study area $j$. $b_{0ij}$ is allowed to vary between particpants at Level 2 around $\beta_{00j}$ (mean of $y_{tij}$ across the time points for all particpants in a study area $j$). $\beta_{00j}$ is allowed to vary between areas at Level 3 around the overall mean $\gamma_{000}$. The within-particpant change in the risk marker was operationalised by entering the *time metric* (i.e., measurement year, t=0, 5, or 12 in the study) at Level 1 and allowing its coefficient (slope of time) to vary at Level 2. The random slope of the time metric ($b_{1i}$) represents the linear change in the risk marker for one time unit incease for particpant $i$ (i.e., annual change in the risk marker). The time-specifc residual is $e_{ti}$, and the particpant-specific random errors are $u_{0ij}$ and $u_{1i}$, and the area-specific random error is $v_{00j}$. By substituting Level 3 and Level 2 equations in the Level 1 equation, a single equation model was estimated. The estimated value of the regression coefficient $\boldsymbol{\beta}_{\boldsymbol{11}}$ and its confidence interval were used to report the relationship between walkability and the annual change in the cardio-metabolic risk marker.
